# Genome-wide identification and comprehensive analysis of microRNAs and phased small interfering RNAs in watermelon

**DOI:** 10.1186/s12864-018-4457-8

**Published:** 2018-05-09

**Authors:** Li Liu, Shuchao Ren, Junqiang Guo, Qingyi Wang, Xiaotuo Zhang, Peiran Liao, Shipeng Li, Ramanjulu Sunkar, Yun Zheng

**Affiliations:** 1 Faculty of Life Science and Technology, Kunming University of Science and Technology, Kunming, 650500 China; 2 Yunnan Key Laboratory of Primate Biomedical Research, Institute of Primate Translational Medicine, Kunming University of Science and Technology, Kunming, 650500 China; 3 Faculty of Information Engineering and Automation, Kunming University of Science and Technology, Kunming, 650500 China; 4 School of Life Sciences, Fudan University, Shanghai, 200433 China; 5 Department of Biochemistry and Molecular Biology, Oklahoma State University, Stillwater, 74078 OK USA

**Keywords:** *Citrullus lanatus* L., High-throughput sequencing, microRNA, PHAS, Phased small interfering RNA (phasiRNA), Bioinformatics

## Abstract

**Background:**

MicroRNAs (miRNAs) are a class of endogenous small non-coding RNAs involved in the post-transcriptional gene regulation and play a critical role in plant growth, development and stress responses. Watermelon (*Citrullus lanatus* L.) is one of the important agricultural crops worldwide. However, the watermelon miRNAs and phasiRNAs and their functions are not well explored.

**Results:**

Here we carried out computational and experimental analysis of miRNAs and phased small interfering RNAs (phasiRNAs) in watermelon by analyzing 14 small RNA profiles from roots, leaves, androecium, petals, and fruits, and one published small RNA profile of mixed tissues. To identify the targets of miRNAs and phasiRNAs, we generated a degradome profile for watermelon leaf which is analyzed using the SeqTar algorithm. We identified 97 conserved pre-miRNAs, of which 58 have not been reported previously and 348 conserved mature miRNAs without precursors. We also found 9 novel pre-miRNAs encoding 18 mature miRNAs. One hundred and one 21 nucleotide (nt) PHAS loci, and two hundred and forty one 24 nt PHAS loci were also identified. We identified 127 conserved targets of the conserved miRNAs and TAS3-derived tasiRNAs by analyzing a degradome profile of watermelon leaf.

**Conclusions:**

The presented results provide a comprehensive view of small regulatory RNAs and their targets in watermelon.

**Electronic supplementary material:**

The online version of this article (10.1186/s12864-018-4457-8) contains supplementary material, which is available to authorized users.

## Background

Plant small RNAs (sRNAs), with 21 to 24 nucleotides (nt), play crucial roles in a variety of biological processes, including development, stress responses, defense and epigenetic modifications [1–3]. Based on their origin and biogenesis, small RNAs in plants can be divided into two main categories: microRNA (miRNA) and small interfering RNA (siRNA). The primary transcripts of MIR genes are transcribed by RNA polymerase II [4]. Such primary miRNA transcripts form typical hairpin-like structure that are cleaved twice by Dicer Like protein (DCL1) in the nucleus to excise a miRNA:miRNA* duplex [5]. Then, the duplex is exported to cytoplasm [6]. In the cytoplasm, miRNAs are loaded into an RNA-induced silencing complex (RISC) that normally contains an Argonaute (AGO) protein, and guide the RISC to cause site-specific cleavage or repression of the mRNA targets [7, 8]. The other strand of the duplex, i.e., miRNA*, is degraded [9]. Most plant miRNAs negatively regulate their target genes through homolog based mRNA cleavage or translation inhibition at post-transcriptional level [3, 6, 10], however some miRNAs, especially metazoan miRNAs, may activate their targets [11–13].

In addition to miRNAs, small interfering RNAs (siRNAs) also have important functions in plants. Especially, phasiRNA is a class of secondary siRNAs that are generated precisely in 21 or 24 nt phased pattern initiated at a specific position due to miRNA guided activity. A type of phasiRNAs are *trans*-acting siRNAs (tasiRNAs) because they repress their target transcripts from other loci of the genome at post-transcriptional level. The primary transcripts of tasiRNAs are non-coding and used to generate double strand RNAs (dsRNAs) by RDR6 (RNA-dependent RNA polymerase 6) [14]. The dsRNAs are then cleaved by DCL4 to form phased 21 nt segments [15–17] or by DCL5 to form 24 nt phased segments in rice [18, 19]. The precise phasing of tasiRNAs is guided by miRNAs [15] through either two [20] or one [18, 21–24] miRNA binding site on the primary tasiRNA transcripts. Four families of tasiRNA loci, named TAS1 to TAS4, have been identified in *Arabidopsis thaliana* [15, 17, 25]. Recent studies suggest that some coding genes, especially PPR [17, 24, 26], NB-LRR disease resistance proteins [24, 27–31], MYB transcription factors [24, 32, 33], also generate phasiRNAs, and their corresponding loci are called as PHAS loci [27]. These PHAS loci are also triggered by one or two miRNA binding sites [14, 27]. *TAS3* derived tasiARFs are the only phasiRNAs that have been validated to target ARF genes in *trans* [15, 20, 24]. The functions of most phasiRNAs are still largely unknown [14].

Watermelon is one of the 20 most important agricultural crops worldwide [34]. Although watermelon is economically important, only a few studies [34–38] have paid attention on the miRNAs in watermelon, and there are still no studies about phasiRNAs in watermelon.

To systematically investigate the miRNAs and phasiRNAs in watermelon, we sequenced 14 small RNA profiles from 5 tissues, i.e., roots, leaves, androecium, petals, and fruits. Comprehensive computational analyses of these small RNA profiles lead to identification of 97 pre-miRNAs of which 58 have not been reported, 101 PHAS loci encoding 21 nt phasiRNAs (including a new TAS3 locus), and 241 PHAS loci encoding 24 nt phasiRNAs. A degradome profile of watermelon leaf was generated for identifying targets of miRNAs and phasiRNAs. Totally, 127 conserved targets for conserved miRNAs and TAS3-derived tasiRNAs were identified in the analysis. These results significantly enhanced our understanding of small RNA guided gene regulations in watermelon.

## Methods

### Materials and small RNA sequencing profiles

The watermelon plants were grown in the greenhouse of Faculty of Life Science and Technology, Kunming University of Science and Technology (24° 51 ^′^ 0 ^″^ N, 102° 52 ^′^ 2 ^″^ E, altitude 1835 m), Yunnan, China. During the experiments, the daily average temperature was 25 °C, the daily maximum difference in temperature was 10 °C and humidity was 60-80%. Fourteen samples from five tissues, i.e., root, leaf, petal, androecium and pre-mature fruit, of watermelon plants without treatments were collected and frozen using liquid nitrogen immediately (Additional file 1: Supplementary Table S1). The 14 samples were stored at -80 °C until RNAs were extracted. Total RNAs were extracted from samples using the Trizol reagent according to the manufacturer’s protocol. The integrities of the RNAs were checked using an ultraviolet spectrophotometer (Hoefer, MA, USA), based on the ratio of the optical density at 260 nm to that at 280 nm (OD260/280) and were also assessed by electrophoresis in a denaturing formaldehyde agarose gel, based on visual comparison of the 18S and 28S ribosomal RNAs. The RNA samples with OD260/280 between 1.8 and 2.0 were checked for the total quantities. Only samples with at least 20 *μ*g were chosen for preparation of sRNA sequencing libraries. 20 *μ*g total RNAs dissolved in 35 *μ*l were delivered to the sequencing facility. The small RNAs of the samples were isolated from total RNAs and were sequenced using Illumina HiSeq 2000 sequencer. The obtained small RNA profiles had been deposited to NCBI under GEO the accession number, GSE102030. The qualities of the obtained sRNA profiles were evaluated with the FASTQC program (https://www.bioinformatics.babraham.ac.uk/projects/fastqc/) (in Additional file 2: Figure S1).

### Identification of conserved and novel miRNAs

The computational pipelines for analyzing the obtained small RNA profiles were shown in supplementary materials (Additional file 2: Figure S2).

The mRNA and genome sequences of watermelon were downloaded from Cucumber Genome Database (http://cucurbitgenomics.org/). Watermelon (97103) genome and annotation (v6) were downloaded.

Our computational methods for analyzing the small RNA libraries was reported previously [39]. In brief, small RNAs were extracted after removing raw reads with more than 5 low scored nucleotides (< 25) and 3’ adaptor sequences of the remaining reads (see Additional file 2: Figure S2d). Unique small RNAs were obtained after eliminating redundant sequences. The unique sRNAs were aligned to genome and mRNAs of watermelon to calculate the numbers of reads and unique sequences mapped to the genome and mRNAs. Then unique sequences mapped to known non-coding RNAs (rRNAs, tRNAs, snRNAs, snoRNAs) and repeats were removed from unique RNAs by aligning to databases Rfam (r11) [40], NONCODE (v3.0) [41], Silva [42], The TIGR Plant Repeat Databases [43] and Repbase (r20) [44] using SOAP2 [45]. Remaining reads were mapped to the miRBase database (r21) [46] to calculate the frequencies of conserved miRNAs. The numbers of reads that were aligned to different types of molecules were counted and summarized (see Additional file 1: Table S2).

After this step, the remaining unique reads were aligned to genome to predict novel miRNAs. The surrounding sequences (down- and up-stream) were extended by 300 bp which were used to predict fold-back structure using RNAfold [47]. The structures with at least 18 paired nucleotides and a folding energy of smaller than -40 Kcal/mol were kept as putative precursors for further analysis. The small RNA reads were mapped to the obtained putative precursors. Finally, novel miRNA identification and annotation were strictly based on appearance of miRNA* and predictable fold back structures for the miRNA precursor sequences, as suggested by Meyers et al. [48].

As described previously [39], mature miRNA sequences from all plant species were downloaded from the miRBase (v21) [46] from which unique miRNA sequences were obtained (see Additional file 2: Figure S2a). Next, these unique miRNA sequences were used as queries against the watermelon genome using BLASTN to predict known miRNA homologs in watermelon that were not represented in small RNA libraries. Hits with no more than two mismatches were identified and the flanking regions (80nt, 130nt, 180nt down stream and upstream) to the mapped mature miRNAs were isolated and used to predict fold-back structures using the RNAfold. The predicted fold-back structures were examined for the presence of miRNA on the same arm of the hairpin as the known family members from other plants. These precursor sequences were further evaluated by MIRcheck and selected candidates that have ≤ 5 mismatches, ≤ 2 bulged or asymmetrically unpaired nucleotides, and ≤ 3 continuous mismatches within the mature miRNA.

We compared the identified conserved miRNAs with those reported previously [34–38]. If the sequences of a mature miRNA was the same as reported earlier, the conserved miRNA was named the same as reported previously. The remaining conserved miRNAs were named by using upper case MIR followed by the family name, and alphabetical letters in lower case if these have not been reported earlier.

### Analysis of miRNA expression patterns in different tissues

The raw frequencies of mature miRNAs for samples of different tissues were compared with edgeR [49]. miRNAs with multiple-test corrected *P*-values of smaller than 0.05 were regarded as significantly deregulated miRNAs in the different tissues.

miRNA with abundances of at least 5 RPTM (Reads Per Ten Million sequencing tags) in at least two of the 14 samples and standard deviation of at least 1 were selected to perform hierarchical clustering. The normalized RPTM values plus one were log scaled to calculate correlation coefficient (CC) values between samples. Then the CC values were applied to the pheatmap function in the pheatmap library in R to perform hierarchical clustering. miRNAs with average abundances of at least 5 RPTM in the 14 samples were used to perform Principle Component Analysis (PCA). Log-scaled normalized RPTM values plus one were applied to the prcomp function in the psych library in R to perform PCA.

### Identification of TAS3 loci in watermelon

As done previously [50], TAS3 derived tasiRNAs, siRNAs that target the ARF family genes, in Arabidopsis and rice were collected. Then, these tasiRNAs were aligned to the watermelon genome by allowing at most 2 mismatches. The neighboring sequences of the matched loci were cut out to include 250 nt from both 5’ and 3’ sides. Then we examined the typical miR390 complementary sites around the conserved tasiRNAs by predicting the miR390 sites on the cut-out sequences.

### Identification of PHAS loci in watermelon

To predict PHAS loci and phasiRNAs, we used one more small RNA profile of mixed watermelon tissues in our previous work [35] in addition to 14 small RNA profiles generated in the current study. This sRNA profile was downloaded from the from the NCBI GEO database with the accession number GSM936361. This profile and the combined file of the 14 sRNA profiles generated in this study were used to predict PHAS loci and phasiRNAs, respectively.

The PHAS loci and phasiRNA were predicted as described previously [39, 51]. The unique sequences in the small RNA libraries were mapped to Repbase (r20) [44] and The TIGR Plant Repeat Databases [43] to remove sRNAs mapped to repeats, then the remaining sRNAs were mapped to the genome of watermelon using SOAP2 [45] (see Additional file 2: Figure S2b). A self-written program was used to scan the genome and cDNA sequences using a window of 210 nt or 240 nt (ten 21 nt or 24 nt) respectively. A two-nucleotide positive offset was used to calculate the positions of siRNAs on the anti-sense strand because the existence of two-nucleotide over-hang at the 3’-end of siRNA duplex [17, 24, 26, 27]. Then a *P*-value was calculated for each of the windows using a modified version of methods in [26],

1 $$ P(X=k) = \sum_{X=k}^{m}\frac{{20m \choose n-k}{m \choose k}}{{21m \choose n}},  $$

where *n* was the number of unique 21 nt (or 24 nt) sRNAs mapped within a window, *k* was the number of phased unique 21 nt (or 24 nt) sRNAs within the window, and *m* was the number of phases. In this study, *m* was set to 10.

And a phase score was calculated for each position of the genome and cDNA sequences using the method in [52]. For a window started at a position with more than three phased unique sRNAs, i.e., when *k*≥3,

2 $$ PhaseScore = \text{ln}\left(1 + 10 \times \frac{\sum_{i = 1}^{m}P_{i}}{1+\sum_{i=1}^{m}U_{i}}\right)^{k - 2},  $$

where *P*_*i*_ was the number of phased reads at the *i*th phase from the position, *U*_*i*_ was the number of non-phased reads at the *i*th phase from the position, and *m* was the number of phases in the window, and *k* was the number of unique phased siRNAs in the window. *m* was 10 in this study.

The window with a *P*-value less than 0.05 was extended 100 bp at both 5’- and 3’-ends, then the overlapped windows were merged. The *P*-values of the merged windows were used to calculate the false positive rates using the method in [53]. The merged windows with a maximal phase scores of larger than pre-determined threshold and multiple test corrected *P*-values of smaller than 0.05 were reported as PHAS loci. The predicted PHAS loci were named with its chromosome and a unique serial number for each chromosome. “P21” and “P24” were added at the beginning of the predicted PHAS loci encoding 21 and 24 nt phasiRNAs. The neighboring PHAS loci were predicted as PHAS clusters if the distances between individual PHAS loci were smaller than 2000 base pairs. The phased siRNAs of the predicted PHAS loci were reported as phasiRNAs. The phasiRNAs of a PHAS loci were named by adding siR and a serial number to the name of the PHAS loci.

### Degradome sequencing

A leaf sample of a watermelon plant grown in greenhouse at Kunming University of Science and Technology, Kunming, Yunnan, China, was frozen in liquid nitrogen immediately after harvesting. The total RNA from the leaf of a watermelon plant was extracted using the Trizol reagent according to the manufacturer’s protocol. The integrity of the RNA was checked with an ultraviolet spectrophotometry and 2100 BioAnalyzer. The degradome of polyadenylated transcripts was sequenced using Illumina HiSeq 4,000 sequencer. The degradome sequencing profile had been deposited into the NCBI GEO database under the accession number, GSE102030.

The quality of the obtained degradome profile was evaluated with the FASTQC program. The obtained degradome profile was processed with a self-developed pipeline (see Additional file 2: Figure S2c). Firstly, the raw degradome sequencing profile was filtered to remove low quality reads that have low scored nucleotides (< 20) in the first 20 nucleotides. Next, the first 20 nucleotides in remaining reads were used in later analysis. The unique sequences were obtained and the frequencies of the unique sequences were calculated. Then, similar to sRNA profiles, unique sequences were aligned to genome and mRNAs of watermelon, Rfam (r11) [40], NONCODE (v3.0) [41], Silva [42], The TIGR Plant Repeat Databases [43] and Repbase (r20) [44] using SOAP2 [45] to calculate the numbers of reads and unique sequences mapped to different categories of molecules as well as repeats. The SOAP2 alignment result of mapping degradome reads to watermelon mRNAs was used to predict miRNA and siRNA targets, as detailed below.

### Identification of miRNA/siRNA targets in watermelon

The targets of miRNAs and tasiRNAs were predicted with the SeqTar algorithm [54]. For conserved miRNAs and tasiRNAs, the targets that had less than or equal to four mismatches were used for further analysis. For novel miRNAs, only targets with at least 1 valid read and less than 4 mismatches were used.

The targets of phasiRNAs were also predicted with the SeqTar algorithm. Only targets that have no mismatches or at least one valid degradome read and less than or equal to 3 mismatches were used for further analysis.

### Identifying miRNA complementary sites on PHAS loci

The SeqTar algorithm was used to predict miRNA complementary sites on the original transcripts of PHAS loci. For conserved miRNAs, the targets that have at least one valid read, i.e., read started at the 9th to 11th positions of a miRNA binding site (as defined in [54]), or targets that have less than 4 mismatches were used for further analysis.

## Results and discussion

### High-throughput sequencing profiles of watermelon Small RNAs

We generated 14 small RNA libraries from the pooled RNA isolated from five different tissues (root, leaf, petal, androecium and premature fruit). Plants were grown under greenhouse conditions without special treatments. The small RNAs of these 14 samples were sequenced using Illumina HiSeq 2000 sequencer. Initially the 3’ adapter from the raw reads was trimmed and small RNAs ranging between 18–30 nucleotides were extracted. Upon sequencing of these 14 small RNA libraries, we obtained approximately 20 million reads for each of the libraries. Totally, we obtained 280,698,505 total reads represented by 60,516,813 unique small RNAs (Additional file 1: Table S1). After examining the scores per nucleotides with FASTQC, the qualities of the obtained sRNA sequencing profiles were generally good with scores above 30 for the first 25 nt (see Additional file 2: Figure S1), which include most small RNAs with 21 to 24 nt. The small RNA sequences were aligned to mRNAs of watermelon, precursors of miRNAs in the miRBase (v21), other non-coding RNAs besides miRNAs, repeat elements and genome of watermelon with SOAP2 (see details in Figure S2 and in Materials and methods). Most reads could be aligned to the genome of watermelon (see Additional file 1: Table S2), suggesting high qualities of the obtained sRNA profiles. The total reads of 21 nt and 24 nt are over-represented while the unique reads of 23 nt and 24 nt are over-represented in all four libraries (Additional file 2: Figure S3). The 21 nt reads and unique sequences represents the only peak for those sequences mapped to the miRNA precursors (Additional file 2: Figure S3), consistent with the lengths of mature plant miRNAs.

### Identification of conserved miRNAs and their expression patterns in different tissues

**Fig. 1:**
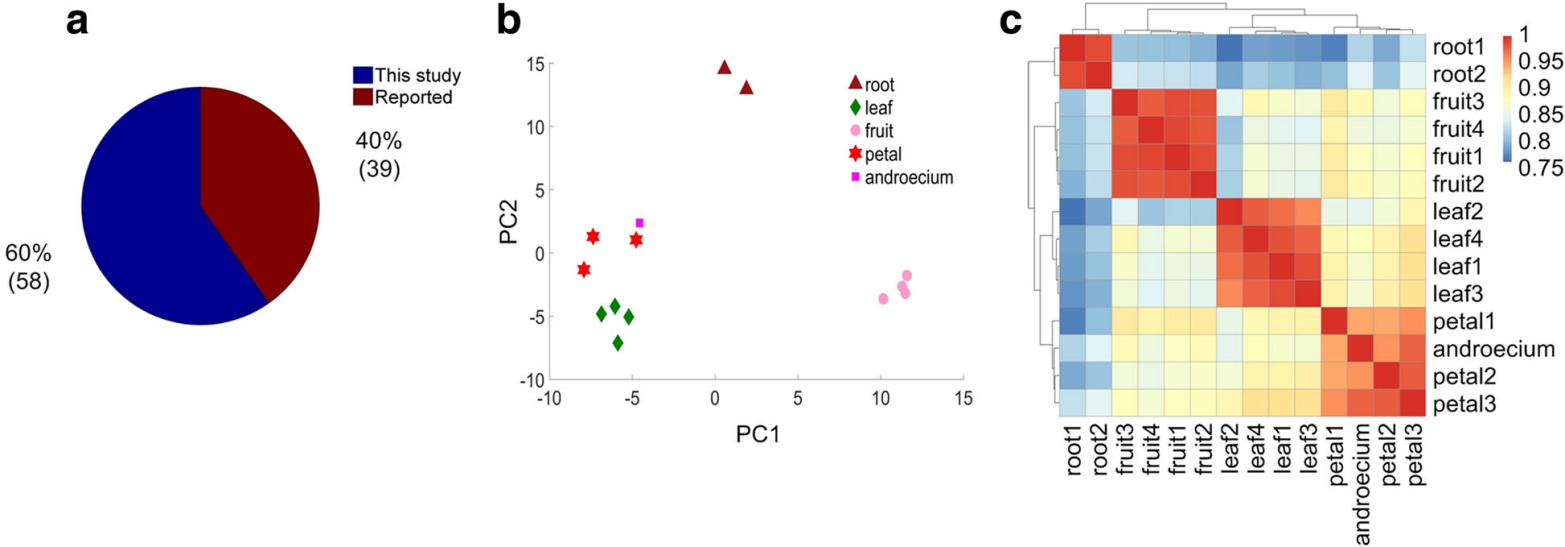
The conserved miRNAs in watermelon and their expression patterns in different tissues. **a** The number of pre-miRNAs identified in this study and that reported in literature. **b** The PCA analysis of miRNA expression profiles in different tissues. **c** The hierarchical clustering of miRNA expession profiles in different tissues

By aligning mature miRNAs from other species to watermelon genome (see details in Figure S2 and in Materials and methods), we identified 97 pre-miRNAs of which 58 have not been reported previously (Fig. 1a and Additional file 1: Table S3). We also found 348 mature miRNAs without precursor by aligning the sRNA profiles to the mature miRNAs in the miRBase (v21) (Additional file 1: Table S3). When compared to two model species, *Arabidopsis thaliana* and rice, we found 80 members that belong to 20 miRNA families that are highly conserved (Additional file 1: Table S4).

Because we have sequenced small RNAs from five different tissues of watermelon, it is feasible to assess the differences in miRNA expression patterns in root, leaf, petal, androecium and premature fruit. We performed Principle Component Analysis and Hierarchical Clustering based on the normalized frequencies of mature miRNAs (Additional file 1: Table S5). The samples from the same tissues were clustered together and samples from different tissues were clearly differentiated (Fig. 1b). We also found the strong correlation among the samples of the same tissues, meanwhile samples from different tissues have much lower correlation coefficient values (Fig. 1c). These results suggest that miRNAs in different tissues have important functions and perhaps involve in different physiological processes in different tissues.

We also compared the abundances of conserved miRNAs for samples from root, leaf, petal, and pre-mature fruit with edgeR [49] to identify deregulated miRNAs in different tissues. This analysis has identified 87 to 153 deregulated miRNAs between the compared tissues (Additional file 1: Table S6). For examples, MIR156i_5p has higher expression levels in leaves than in the roots, petals, and fruits; MIR319g has higher expression level in petals than in leaves; MIR167f-5p has higher expression level in petals than in roots (Additional file 1: Table S7).

We also identified 9 putative novel miRNAs in watermelon (Additional file 1: Table S8). Although miRNA and miRNA* were identified for these miRNAs, their expression levels are generally very low when being compared to conserved miRNAs, suggesting that these novel miRNAs may be non-functional or not functionally important.

### Identifying miRNA targets in watermelon

To identify the miRNA targets, we generated a degradome profile with over 23 million reads, of which most could be aligned to watermelon genome (Additional file 1: Table S9). The nucleotide scores of the obtained degradome were of high quality since most nucleotides had scores above 35 (Additional file 2: Figure S4). We identified targets of conserved miRNA by analyzing the obtained degradome profile of watermelon leaf using the SeqTar algorithm [54]. This analysis revealed 127 conserved targets for conserved miRNA families and TAS3 generated tasiARFs (Additional file 2: Tables S10 and S11).

**Fig. 2:**
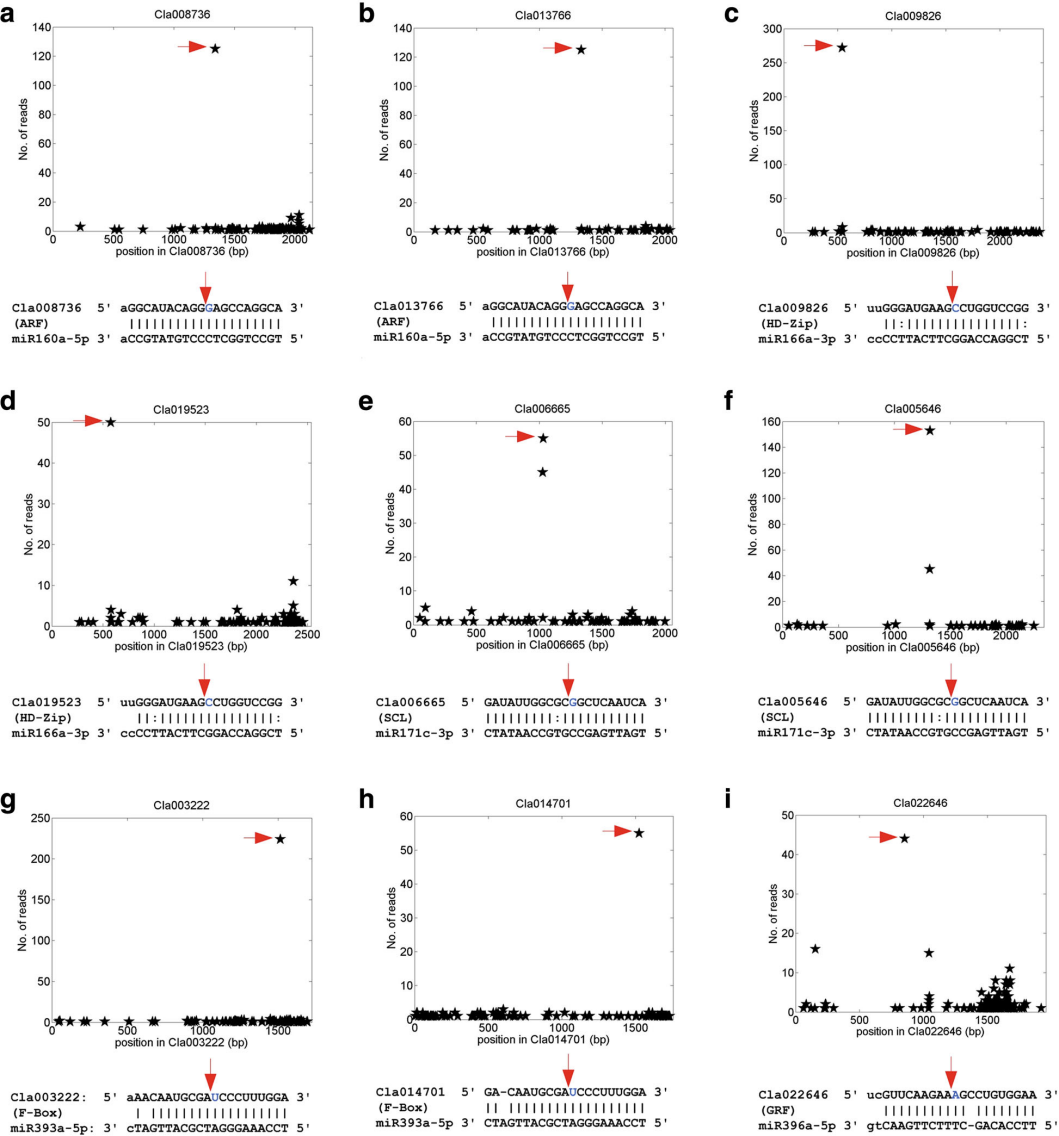
Some of the identified conserved miRNA targets. The x-axis is the position on the transcript, and y-axis is the number of reads detected from a position. The arrows in the upper parts correspond to the positions pointed by the arrows of the same colors in the lower parts. **a** miR160a-5p:Cla008736, an ARF gene. **b** miR160a-5p:Cla013766, an ARF gene. **c** miR166a-3p:Cla009826, an HD-Zip gene. **d** miR166a-3p:Cla019523, an HD-Zip gene. **e** miR171c-3p:Cla006665, a GRAS/SCL transcription factor gene. **f** miR171c-3p:Cla005646, a GRAS/SCL transcription factor gene. **g** miR393a-5p:Cla003222, an F-box gene. **h** miR393a-5p:Cla014701, an F-box gene. **i** miR393a-5p:Cla003222, a GRF gene

Some of the conserved miRNA targets are shown in Fig. 2. The miR160 family targets 5 ARF genes (in Additional file 1: Table S10), of which two are shown in Fig. 2a and 2b. The miR166 family targets five HD-Zip transcription factors (in Additional file 1: Table S10), of which two are shown in Fig. 2c and 2d. The miR171 family targets eight SCL transcription factors (in Additional file 1: Table S10), of which two are shown in Fig. 2e and 2f. The miR393 family targets 8 F-box genes (in Additional file 1: Table S10), of which two are shown in Fig. 2g and 2h. The miR396 family targets 8 GRF transcriptional factors (in Additional file 1: Table S10), of which one is shown in Fig. 2i. Several other conserved miRNAs also cleaved their targets with significant number of valid degradome reads (see Additional file 2: Figure S5).

In additional to those 127 conserved targets (in Additional file 1: Table S10), conserved miRNA may regulate non-conserved targets as well (Additional file 1: Table S12). Only 48 putative targets were identified for novel miRNAs and most of these targets have no or very limited valid reads at the identified miRNA complementary sites (Additional file 1: Table S13), again suggesting these novel miRNAs might be non-functional.

### Identification of TAS3 loci in watermelon

**Fig. 3:**
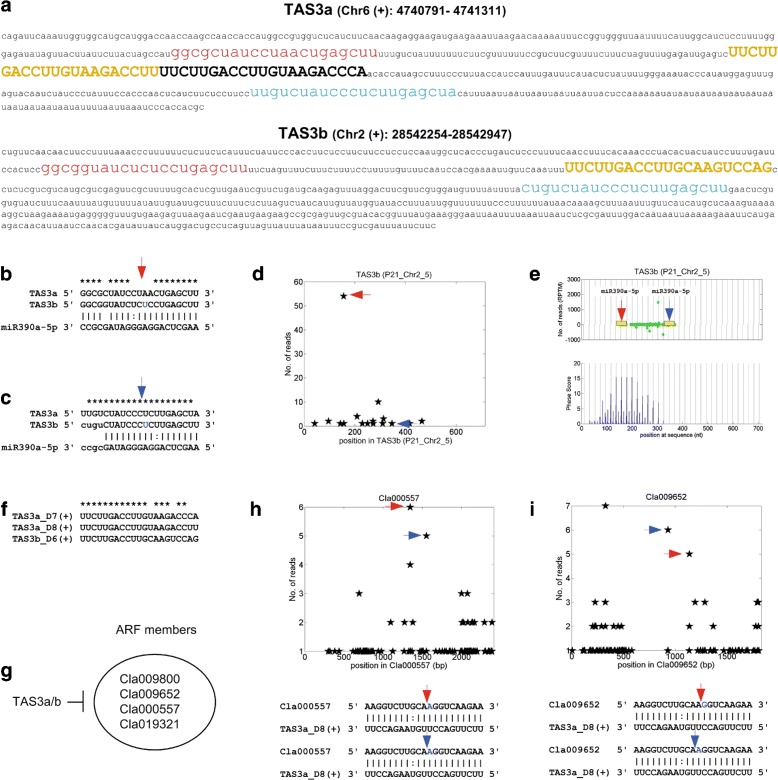
The TAS3 loci identified in watermelon. **a** The sequence and genomic locus of TAS3a and TAS3b. The red and blue region are the 5’ and 3’ miR390 complementary sites. The yellow and black regions in upper cases are the tasiRNAs that target ARF genes. **b** The 5’ miR390 site on TAS3a and TAS3b. **c** The 3’ miR390 site on TAS3a and TAS3b. **d** The distribution of degradome reads on TAS3b transcript. The two positions pointed by red and blue arrows correspond to the 5’ and 3’ miR390 site shown in Part **b** and **c**, respectively. **e** The distribution of small RNA reads in the small RNA profile of mixed tissues (GSM936361) on TAS3b. **f** The tasiRNAs that target ARF genes in TAS3a and TAS3b, named by following the scheme in [15]. **g** Four ARF genes that are targeted by TAS3 derived tasiRNAs. **h** The T-plot of and complementary sites of TAS3a_D8(+):Cla000557. **i** The T-plot of and complementary sites of TAS3a_D8(+):Cla009652. In Part **h** and **i**, the x-axis is the position on the transcript, and y-axis is the number of reads detected from a position. The arrows in the upper parts correspond to the positions pointed by the arrows of the same colors in the lower parts

We previously found one TAS3 in watermelon by analyzing ESTs (Expressed Sequencing Tags) of watermelon [35]. In this study, the previously identified TAS3 loci was found on chromosome 6 of watermelon, named as TAS3a (Fig. 3a). We found an additional TAS3 locus in this study, named as TAS3b, on chromosome 2 of watermelon in this study (Fig. 3b). Both of these two loci have typical miR390 sites beside the tasiRNA generation region (Fig. 3b to c). It is interesting that both the 5’ and the 3’ miR390 site on TAS3b could induce cleavage of TAS3b transcripts (Fig. 3d), and the 5’ miR390 site is the major site that induces more cleavages on TAS3b transcripts. The 10 to 11 positions of 5’ miR390 sites are complementary on TAS3b, but not matched on TAS3a (Fig. 3b), which might be the reason that the 5’ miR390 site on TAS3b is cleavable, but non-cleavable on TAS3a. TAS3b shows a clear phased siRNA generation patterns in one of the sRNA profiles used (Fig. 3e). TAS3a and TAS3b encode two and one tasiRNAs, respectively (Fig. 3a and f). These three tasiRNAs target 4 ARF genes (Fig. 3g). Two ARF genes were cleaved by these TAS3-derived tasiRNAs at two different sites as revealed by the degradome analysis (Fig. 3h and i).

Typically, the 3’ miR390 is the only or major site that could induce cleavage on TAS3 transcripts [20]. But Xia et al. [55] noticed that many spruce TAS3 genes include a cleavable 5’ miR390 site. Thus, our results indicate that TAS3 loci of other species may also have cleavable 5’ miR390 sites in addition to spruce.

### PHAS loci in watermelon

**Fig. 4:**
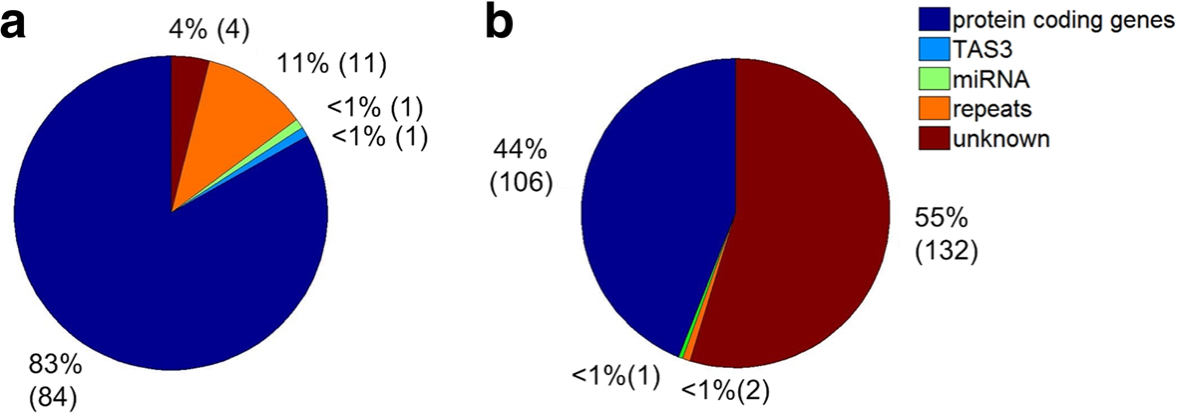
The categories of predicted PHAS loci. **a** The categories of 21 nt PHAS loci. **b** The categories of 24 nt PHAS loci

The combined sRNA profiles of 14 samples in this study and one additional sRNA profile downloaded from NCBI GEO database were aligned to watermelon genome using SOAP2 [45], respectively. Then, we used a self-written program to predict PHAS loci and phasiRNAs from the alignment results of SOAP2 based on methods described earlier (see Materials and methods for details). We predicted one hundred and one 21nt PHAS loci by using a phase score threshold of 5 and a multiple-test corrected *P*<0.05 (Additional file 1: Table S14). These PHAS loci produced at least four unique twenty-one nt phasiRNAs that were sequenced in the sRNA libraries used in study. We aligned the obtained PHAS sequences to NCBI Nucleotide Collection (nr/nt) database, the coding gene of watermelon and the TIGR Plant Repeat database to obtain putative annotation of the predicted PHAS loci (details are given in Additional file 1: Table S14). These PHAS loci were mainly originated from protein coding genes (Fig. 4a). The second largest category is repeats (as shown in Fig. 4a). One of the predicted 21 nt PHAS loci is a TAS3 locus.

Two hundred and forty one 24 nt PHAS were predicted by using a phase score threshold of 5 and a multiple-test corrected *P*<0.05 (Additional file 1: Table S15). The 24 nt PHAS loci are different from the 21 nt PHAS loci in watermelon, with the largest category belonging to unknown category (Fig. 4b). The second largest category of 24 nt PHAS loci is protein coding genes (Fig. 4b).

**Fig. 5:**
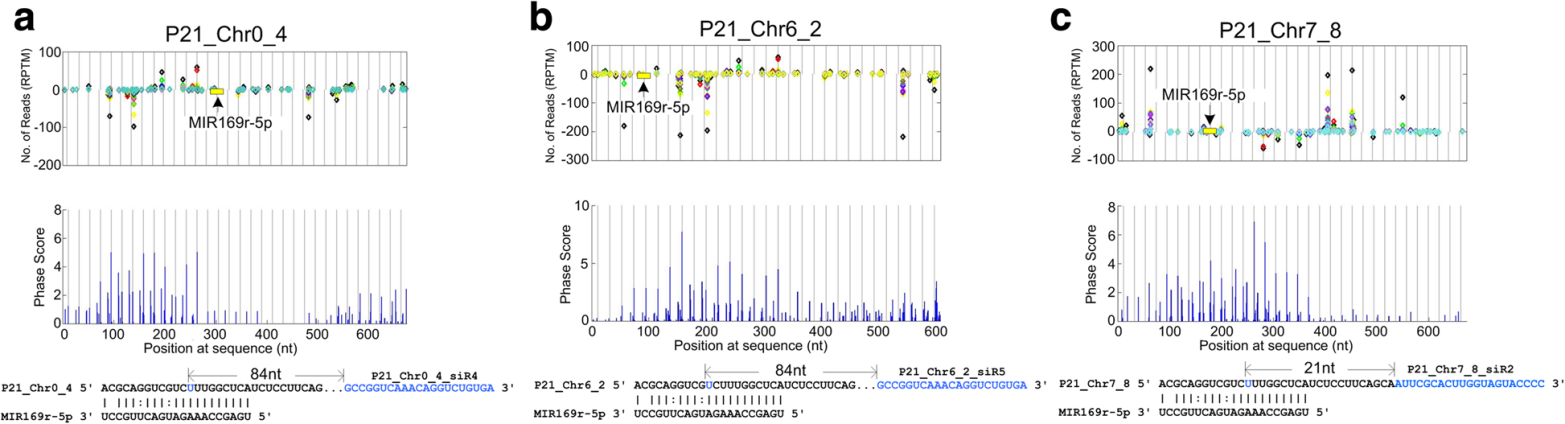
The reads distribution and Phase Scores for three 21 nt PHAS loci potentially triggered by MIR169r-5p. The vertical gray lines with distances of 21 nt are the phased positions from the position with the highest phase scores of the PHAS loci. The yellow boxes in the read distribution panel represent the MIR169r-5p complementary sites. Sites pointed by miRNAs from above and under zero read line means miRNAs complement to the plus and minus strand of the predicted PHAS loci, respectively. The predicted MIR169r-5p complementary sites are shown below the phase score panel. The blue region after the complementary sites are one of the phasiRNAs generated from the PHAS loci. **a** P21_Chr0_4. **b** P21_Chr6_2. **c** P21_Chr7_8

As miRNAs are critical in generation of phasiRNAs, we identified miRNA triggers for these PHAS loci on both strands by using the degradome sequencing profile and the SeqTar algorithm. We found that the MIR169r-5p may trigger the generations of 21 phasiRNAs at three PHAS loci (Fig. 5). All of these three loci are unknown proteins (Additional file 1: Table S14). Except miR390 with 21 nt, most miRNA triggers of 21 nt PHAS loci are 22 nt, such as miR482/miR2118 [14, 19, 27, 56, 57], miR828 [24, 25, 32, 33, 56], and miR7122, miR1509, miR173 [17, 24, 27]. Our results suggested that MIR169r-5p, with 21 nt, potentially triggers the generation of phasiRNAs.

**Fig. 6:**
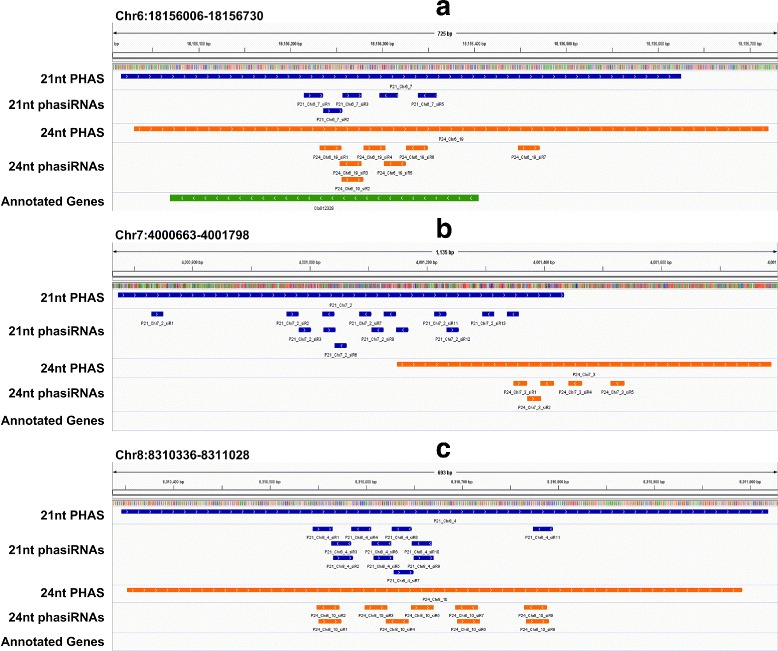
Three loci that generate both 21 nt and 24 nt phasiRNAs in watermelon. The blue and orange lanes represent the 21 nt PHAS/phasiRNAs and 24 nt PHAS/phasiRNAs in watermelon, respectively. **a** P21_Chr6_7/P24_Chr6_19, from an unknown gene locus (Cla012328). **b** P21_Chr7_2/P24_Chr7_3 from an intergenic or un-annotated region. **c** P21_Chr8_4/P24_Chr8_10 from an intergenic or un-annotated region

In several previous studies, we found the some PHAS loci in other species may generate both 21 and 24 nt phasiRNAs [39, 56]. In this study, we found that three PHAS loci may generate both 21 and 24 nt phasiRNAs (Fig. 6). Only the first locus shown in Fig. 6a was annotated as an unknown gene, the other two loci in Figs. 6b and c were intergenic or un-annotated genes.

We did not find miR482 and miR2118 in watermelon, as in previous studies [34–38], suggesting that this miRNA family is non-conserved in watermelon. The miR482/miR2118 family is reported to trigger phasiRNAs by targeting NB-LRR genes in some plant species [24, 27–31, 51]. Overall, our results suggest that the miR482/miR2118 triggered phasiRNA generation pathway is not conserved in curcubits.

### Putative phasiRNAs targets in watermelon

The 101 and 241 PHAS loci encoding 851 and 1227 twenty one and twenty four nt phasiRNAs, respectively (in Additional file 1: Table S16 and S17, respectively).

We found that in addition to some *cis*-targets, where phasiRNAs target their own transcripts, some phasiRNAs may target other genes in *trans* (see Additional file 1: Table S18 and S19 for 21 and 24 nt phasiRNAs, respectively). For examples (see Additional file 2: Figure S6), P21_Chr10_2_siR6 targets Cla021422, a BHLH transcription factor; P21_Chr1_12_siR7 target Cla005420, a photosystem II polypeptide; P21_Chr6_4_siR13 targets Cla006161, a Hydroxycinnamoyl transferase and P21_Chr5_3_siR3 targets Cla018830, a NADPH-cytochrome P450 reductase. These phasiRNAs induce significant cleavages on their corresponding target transcripts, suggesting that they might have *trans*-targets.

## Conclusion

Watermelon is an important plants for economical and agricultural reason. In depth miRNA and PhasiRNA studies have been lacking for this important species. We systematically sequenced 14 small RNA profiles for five different tissues of watermelon, and identified 97 pre-miRNAs, 101 PHAS loci encoding 21 nt phasiRNAs, and 241 PHAS loci encoding 24 nt phasiRNAs. The expression patterns of miRNAs vary in different tissues and show to be very similar for samples of the same tissues. Samples from the same tissues could be corrected clustered based on the expression profiles of miRNAs, suggesting the important functions of miRNAs in different tissues. We also identified one additional TAS3 locus. One hundred and twenty seven targets of conserved miRNAs and TAS3 derived tasiRNAs were identified by analyzing a degradome profile. These results significantly improved our knowledge of sRNA guided gene regulations in watermelon.

## Additional files

Additional file 1 This is an MS Excel file. This file includes 19 supplementary tables. (XLSX 3645 kb)

Additional file 2 This is a pdf file. This file includes 6 supplementary figures. (PDF 2335 kb)

## Abbreviations

AGO: Argonaute
ARF: Auxin response factor
BHLH: Basic helix-loop-helix
BLAST: Basic local alignment search tool
BLASTN: Nucleotide BLAST
CC: Correlation coefficient
cDNA: Complementary DNA
DCL1: Dicer like-1
dsRNAs: Double strand RNAs
ESTs: Expressed sequencing tags
FDR: False discovery rate
GRF: Growth regulatory factors
HD-Zip: Homeodomain leucine zipper family of transcription factors
logFC: log2FoldChange
miRNAs: microRNAs
MYB: Myeloblastosis family of transcription factors
NADPH: Nicotinamide adenine dinucleotide phosphate
NB-LRR: Nucleoside-binding site leucine rich repeat genes
PCA: Principle component analysis
phasiRNAs: Phased small interfering RNAs
PPR: Pentatricopeptide repeats
RDR6: RNA-dependent RNA polymerase 6
RISC: RNA-induced silencing complex
RNA-seq: RNA sequencing
RPTM: Reads Per Ten Million sequencing tags
SCL: Scarecrow-like transcription factor
SeqTar: SEQuencing-based sRNA TARget prediction siRNAs: Small interfering RNAs
SOAP: Short oligonuclotide analysis program
sRNA: small RNA
tasiRNAs: *Trans*-acting small interfering RNAs

## References

[1] Voinnet O (2009). Origin, biogenesis, and activity of plant MicroRNAs. Cell.

[2] Chen X (2012). Small RNAs in development–insights from plants. Curr Opin Genet Dev.

[3] Sunkar R, Li Y-F, Jagadeeswaran G (2012). Functions of microRNAs in plant stress responses. Trends Plant Sci.

[4] Chen X (2009). Small RNAs and their roles in plant development. Annu Rev Cell Dev.

[5] Park W, Li J, Song R, Messing J, Chen X (2002). CARPEL FACTORY, a Dicer homolog, and HEN1, a novel protein, act in microRNA metabolism in *Arabidopsis thaliana*. Curr Biol.

[6] Bartel DP (2004). MicroRNAs: genomics, biogenesis, mechanism, and function. Cell.

[7] Jones-Rhoades MW, Bartel DP, Bartel B (2006). MicroRNAs and their regulatory roles in plants. Annu Rev Plant Biol.

[8] Sunkar R, Zhu J-K (2007). MicroRNAs and short-interfering RNAs in plants. J Integr Plant Biol.

[9] Pasquinelli AE (2012). MicroRNAs and their targets: recognition, regulation and an emerging reciprocal relationship. Nat Rev Genet.

[10] Axtell MJ (2013). Classification and comparison of small RNAs from plants. Annu Rev Plant Biol.

[11] Vasudevan S, Tong Y, Steitz JA (2007). Switching from repression to activation: microRNAs can up-regulate translation. Science.

[12] Vasudevan S (2012). Posttranscriptional upregulation by microRNAs. Wiley Interdiscip Rev RNA.

[13] Xiao M, Li J, Li W, Wang Y, Wu F, Xi Y, Zhang L, Ding C, Luo H, Li Y (2017). MicroRNAs activate gene transcription epigenetically as an enhancer trigger. RNA Biol.

[14] Fei Q, Xia R, Meyers BC (2013). Phased, secondary, small interfering RNAs in posttranscriptional regulatory networks. Plant Cell.

[15] Allen E, Xie Z, Gustafson AM, Carrington JC (2005). microRNA-directed phasing during trans-acting siRNA biogenesis in plants. Cell.

[16] Xie Z, Allen E, Wilken A, Carrington JC (2005). DICER-LIKE 4 functions in trans-acting small interfering RNA biogenesis and vegetative phase change in *Arabidopsis thaliana*. Proc Natl Acad Sci USA.

[17] Howell MD, Fahlgren N, Chapman EJ, Cumbie JS, Sullivan CM, Givan SA, Kasschau KD, Carrington JC (2007). Genome-Wide Analysis of the RNA-DEPENDENT RNA POLYMERASE6/DICER-LIKE4 Pathway in *Arabidopsis* Reveals Dependency on miRNA- and tasiRNA-Directed Targeting. Plant Cell.

[18] Johnson C, Kasprzewska A, Tennessen K, Fernandes J, Nan G-L, Walbot V, Sundaresan V, Vance V, Bowman LH (2009). Clusters and superclusters of phased small RNAs in the developing inflorescence of rice. Genome Res.

[19] Song X, Li P, Zhai J, Zhou M, Ma L, Liu B, Jeong DH, Nakano M, Cao S, Liu C (2012). Roles of DCL4 and DCL3b in rice phased small RNA biogenesis. Plant J.

[20] Axtell MJ, Jan C, Rajagopalan R, Bartel DP (2006). A two-hit trigger for siRNA biogenesis in plants. Cell.

[21] Chen HM, Chen L-T, Patel K, Li YH, Baulcombe DC, Wu SH (2010). 22-Nucleotide RNAs trigger secondary siRNA biogenesis in plants. Proc Natl Acad Sci.

[22] Cuperus JT, Carbonell A, Fahlgren N, Garcia-Ruiz H, Burke RT, Takeda A, Sullivan CM, Gilbert SD, Montgomery TA, Carrington JC (2010). Unique functionality of 22-nt miRNAs in triggering RDR6-dependent siRNA biogenesis from target transcripts in *Arabidopsis*. Nat Struct Mol Biol.

[23] Manavella PA, Koenig D, Weigel D (2012). Plant secondary siRNA production determined by microRNA-duplex structure. Proc Natl Acad Sci.

[24] Xia R, Meyers BC, Liu Z, Beers EP, Ye S, Liu Z (2013). MicroRNA superfamilies descended from miR390 and their roles in secondary small interfering RNA biogenesis in eudicots. Plant Cell.

[25] Rajagopalan R, Vaucheret H, Trejo J, Bartel DP (2006). A diverse and evolutionarily fluid set of microRNAs in *Arabidopsis thaliana*. Genes Dev.

[26] Chen HM, Li Y-H, Wu SH (2007). Bioinformatic prediction and experimental validation of a microRNA-directed tandem trans-acting siRNA cascade in *Arabidopsis*. PNAS.

[27] Zhai J, Jeong D-H, De Paoli E, Park S, Rosen BD, Li Y, González AJ, Yan Z, Kitto SL, Grusak MA (2011). MicroRNAs as master regulators of the plant *NB-LRR* defense gene family via the production of phased, *trans*-acting siRNAs. Genes Dev.

[28] Shivaprasad PV, Chen H-M, Patel K, Bond DM, Santos BA, Baulcombe DC (2012). A microRNA superfamily regulates nucleotide binding site–leucine-rich repeats and other mRNAs. Plant Cell.

[29] Li F, Pignatta D, Bendix C, Brunkard JO, Cohn MM, Tung J, Sun H, Kumar P, Baker B (2012). MicroRNA regulation of plant innate immune receptors. Proc Natl Acad Sci.

[30] Källman T, Chen J, Gyllenstrand N, Lagercrantz U (2013). A significant fraction of 21-nucleotide small RNA originates from phased degradation of resistance genes in several perennial species. Plant Physiol.

[31] Zhu QH, Fan L, Liu Y, Xu H, Llewellyn D, Wilson I (2013). miR482 regulation of NBS-LRR defense genes during fungal pathogen infection in cotton. PloS ONE.

[32] Xia R, Zhu H, An Y-q, Beers EP, Liu Z (2012). Apple miRNAs and tasiRNAs with novel regulatory networks. Genome Biol.

[33] Zhu H, Xia R, Zhao B, An YQ, Dardick CD, Callahan AM, Liu Z (2012). Unique expression, processing regulation, and regulatory network of peach (*Prunus persica*) miRNAs. BMC Plant Biol.

[34] Liu N, Yang J, Guo S, Xu Y, Zhang M (2013). Genome-wide identification and comparative analysis of conserved and novel microRNAs in grafted watermelon by high-throughput sequencing. PloS one.

[35] Jagadeeswaran G, Nimmakayala P, Zheng Y, Gowdu K, Reddy UK, Sunkar R (2012). Characterization of the small RNA component of leaves and fruits from four different cucurbit species. BMC Genomics.

[36] Hu J, Sun L, Zhu Z, Zheng Y, Xiong W, Ding Y (2014). Characterization of conserved microRNAs from five different cucurbit species using computational and experimental analysis. Biochimie.

[37] Li H, Dong Y, Chang J, He J, Chen H, Liu Q, Wei C, Ma J, Zhang Y, Yang J, Zhang X (2016). High-throughput microRNA and mRNA sequencing reveals that microRNAs may be involved in melatonin-mediated cold tolerance in *Citrullus lanatus* L. Front Plant Sci.

[38] Sun Y, Niu X, Fan M (2017). Genome-wide identification of cucumber green mottle mosaic virus-responsive microRNAs in watermelon. Arch Virol.

[39] Zheng Y, Li T, Xu Z, Wai CM, Chen K, Zhang X, Wang S, Ji B, Ming R, Sunkar R (2016). Identification of microRNAs, phasiRNAs and their targets in pineapple. Trop Plant Biol.

[40] Burge SW, Daub J, Eberhardt R, Tate J, Barquist L, Nawrocki EP, Eddy SR, Gardner PP, Bateman A (2013). Rfam 11.0: 10 years of RNA families. Nucleic Acids Res.

[41] Bu D, Yu K, Sun S, Xie C, Skogerbø G, Miao R, Xiao H, Liao Q, Luo H, Zhao G (2012). NONCODE v3. 0: integrative annotation of long noncoding RNAs. Nucleic Acids Res.

[42] Quast C, Pruesse E, Yilmaz P, Gerken J, Schweer T, Yarza P, Peplies J, Glöckner FO (2013). The SILVA ribosomal RNA gene database project: improved data processing and web-based tools. Nucleic Acids Res.

[43] Ouyang S, Buell CR (2004). The TIGR Plant Repeat Databases: a collective resource for the identification of repetitive sequences in plants. Nucleic Acids Res.

[44] Bao W, Kojima KK, Kohany O (2015). Repbase update, a database of repetitive elements in eukaryotic genomes. Mobile DNA.

[45] Li R, Yu C, Li Y, Lam TW, Yiu S-M, Kristiansen K, Wang J (2009). SOAP2: an improved ultrafast tool for short read alignment. Bioinformatics.

[46] Kozomara A, Griffiths-Jones S (2014). miRBase: annotating high confidence microRNAs using deep sequencing data. Nucleic Acids Res.

[47] Hofacker IL (2003). Vienna RNA secondary structure server. Nucleic Acids Res.

[48] Meyers BC, Axtell MJ, Bartel B, Bartel DP, Baulcombe D, Bowman JL, Cao X, Carrington JC, Chen X, Green PJ (2008). Criteria for annotation of plant MicroRNAs. Plant Cell.

[49] Robinson MD, McCarthy DJ, Smyth GK (2010). edgeR: a Bioconductor package for differential expression analysis of digital gene expression data. Bioinformatics.

[50] Jagadeeswaran G, Zheng Y, Li Y-F, Shukla LI, Matts J, Hoyt P, Macmil SL, Wiley GB, Roe BA, Zhang W, Sunkar R (2009). Cloning and characterization of small RNAs from Medicago truncatula reveals four novel legume-specific microRNA families. New Phytol.

[51] Zheng Y, Chen K, Xu Z, Liao P, Zhang X, Liu L, Wei K, Liu D, Li YF, Sunkar R (2017). Small RNA profiles from panax notoginseng roots differing in sizes reveal correlation between mir156 abundances and root biomass levels. Sci Rep.

[52] De Paoli E, Dorantes-Acosta A, Zhai J, Accerbi M, Jeong DH, Park S, Meyers BC, Jorgensen RA, Green PJ (2009). Distinct extremely abundant siRNAs associated with cosuppression in petunia. RNA.

[53] Benjamini Y, Hochberg Y (1995). Controlling the false discovery rate: a practical and powerful approach to multiple testing. J R Stat Soc Ser B Methodol.

[54] Zheng Y, Li Y-F, Sunkar R, Zhang W (2012). SeqTar: an effective method for identifying microRNA guided cleavage sites from degradome of polyadenylated transcripts in plants. Nucleic Acids Res.

[55] Xia R, Xu J, Arikit S, Meyers BC (2015). Extensive families of miRNAs and PHAS loci in norway spruce demonstrate the origins of complex phasiRNA networks in seed plants. Mol Biol Evol.

[56] Zheng Y, Wang S, Sunkar R (2014). Genome-wide discovery and analysis of phased small interfering RNAs in chinese sacred lotus. PLoS ONE.

[57] Zhai J, Zhang H, Arikit S, Huang K, Nan G-L, Walbot V, Meyers BC (2015). Spatiotemporally dynamic, cell-type–dependent premeiotic and meiotic phasiRNAs in maize anthers. Proc Natl Acad Sci.

