# Supplementary material for: Genome-wide identification and comprehensive analysis of microRNAs and phased small interfering RNAs in watermelon
Source: BMC Genomics. 2018 May 9;19(Suppl 2):111. doi: 10.1186/s12864-018-4457-8 (PMC5954288; doi:10.1186/s12864-018-4457-8)
Supplement: Supplementary file 2 — This is a pdf file. This file includes 6 supplementary figures. (PDF 2335 kb) [file 12864_2018_4457_MOESM2_ESM.pdf]

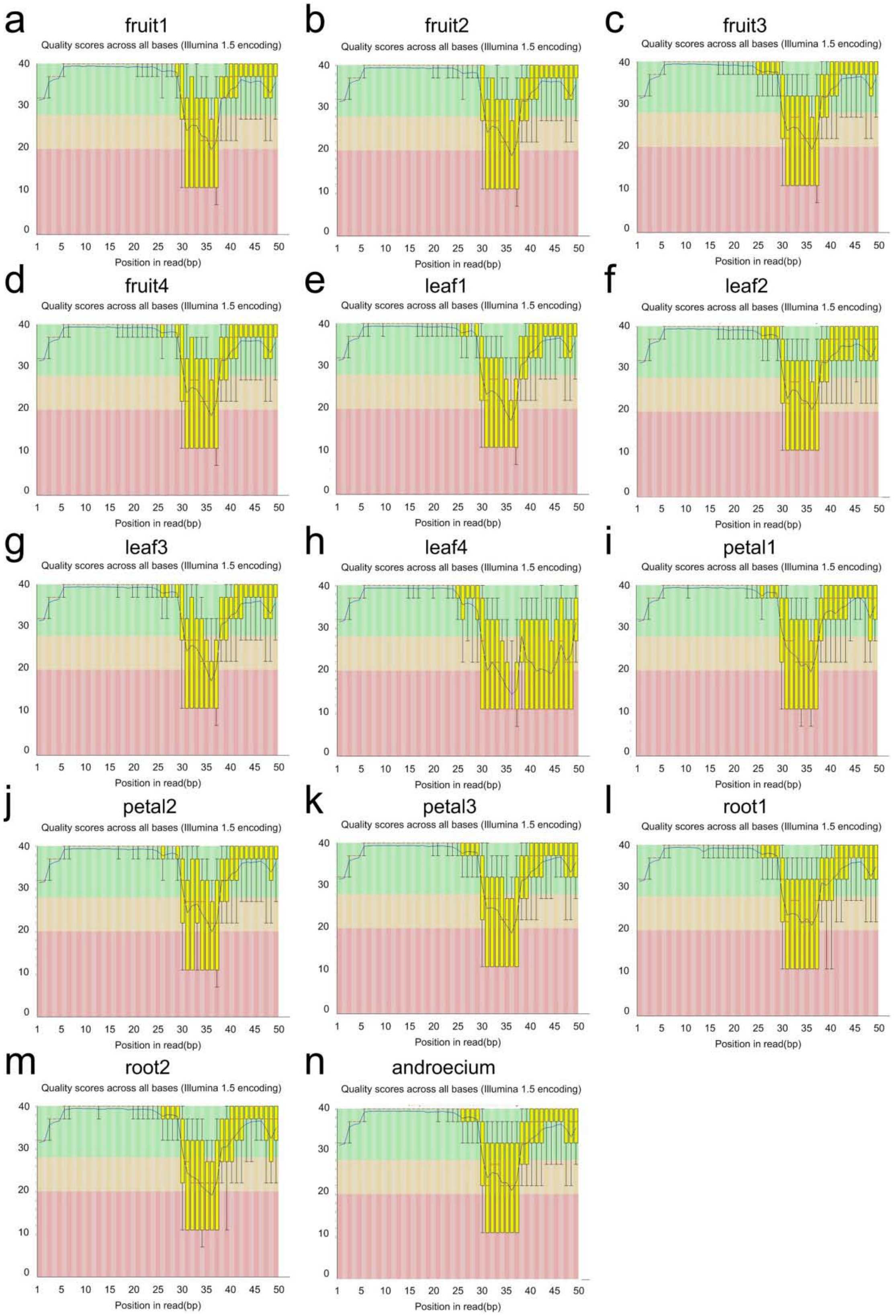

Figure S1. Per base sequencing quality of 14 small RNA sequencing profiles. The horizontal axis is the position of the reads. The vertical is the quality scores of the reads.

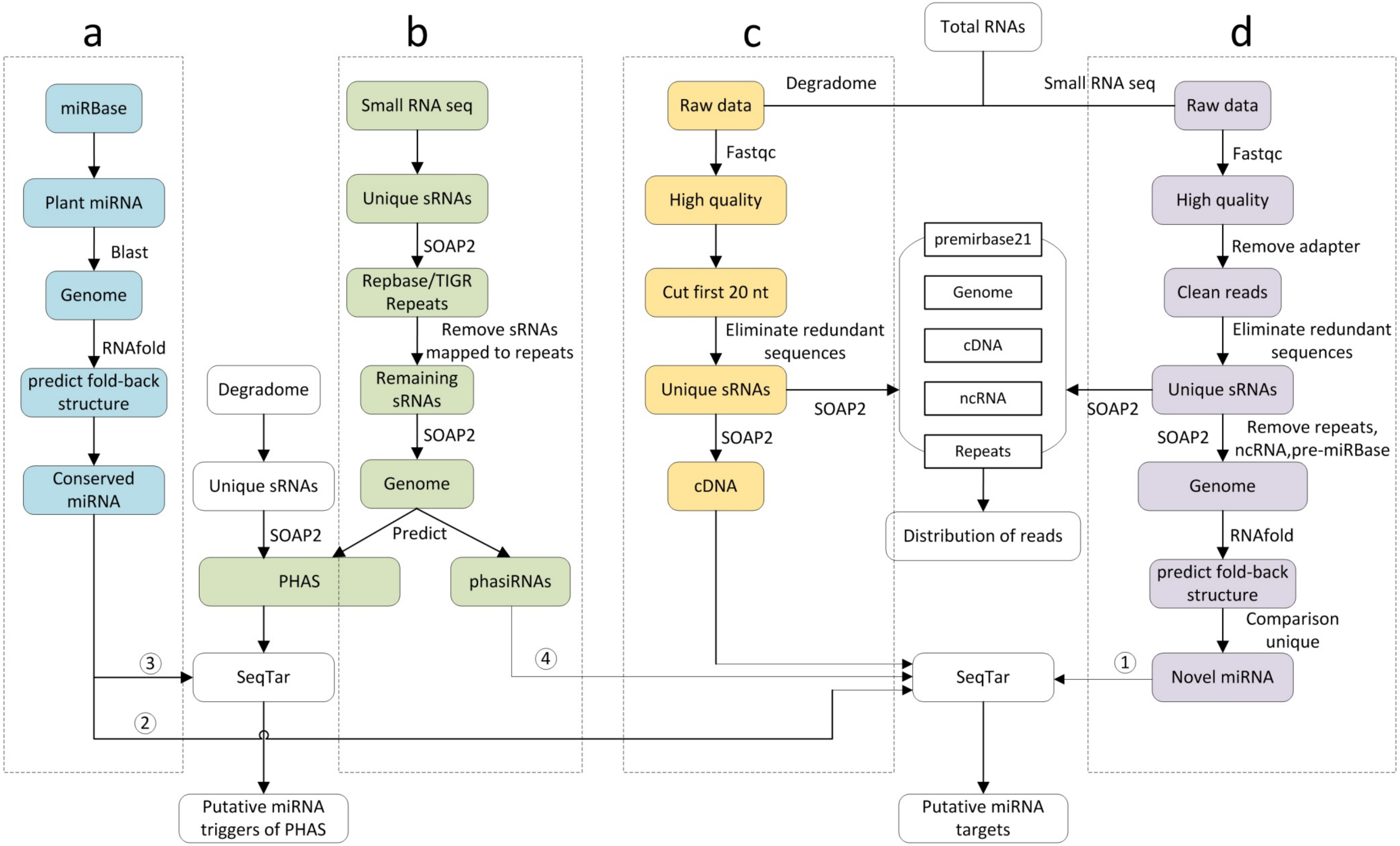

a
b
c
d
Total RNAs
Degradome
Small RNA seq
miRBase
Plant miRNA
Blast
Genome
RNAfold
predict fold-back structure
Conserved miRNA
Small RNA seq
Unique sRNAs
SOAP2
Repbase/TIGR Repeats
Remove sRNAs mapped to repeats
Remaining sRNAs
SOAP2
Genome
Predict
PHAS
phasiRNAs
Degradome
Unique sRNAs
SOAP2
SeqTar
Putative miRNA triggers of PHAS
Raw data
Fastqc
High quality
Cut first 20 nt
Eliminate redundant sequences
Unique sRNAs
SOAP2
cDNA
SOAP2
premirbase21
Genome
cDNA
ncRNA
Repeats
Distribution of reads
SeqTar
Putative miRNA targets
Raw data
Fastqc
High quality
Remove adapter
Clean reads
Eliminate redundant sequences
Unique sRNAs
SOAP2
SOAP2
Remove repeats, ncRNA,pre-miRBase
Genome
RNAfold
predict fold-back structure
Comparison unique
Novel miRNA
1
2
3
4

Figure S2. The steps for analyzing small RNA sequencing profiles and degradome profile in watermelon. (a) The steps for conserved miRNA prediction. (b) The steps for predicting PHAS loci and phasiRNAs. (c) The steps for analyzing degradome sequence profile. (d) The steps for analyzing small RNA-seq profiles and for predicting novel miRNAs. ①②③④ represent four times of SeqTar analysis, i.e., predicting targets of novel miRNAs using novel miRNAs and cDNAs, predicting targets of conserved miRNAs using conserved miRNAs and cDNAs, predicting miRNA triggers of PHAS loci using conserved miRNAs and PHAS sequences, and putative targets of phasiRNAs using phasiRNAs and cDNAs, respectively.

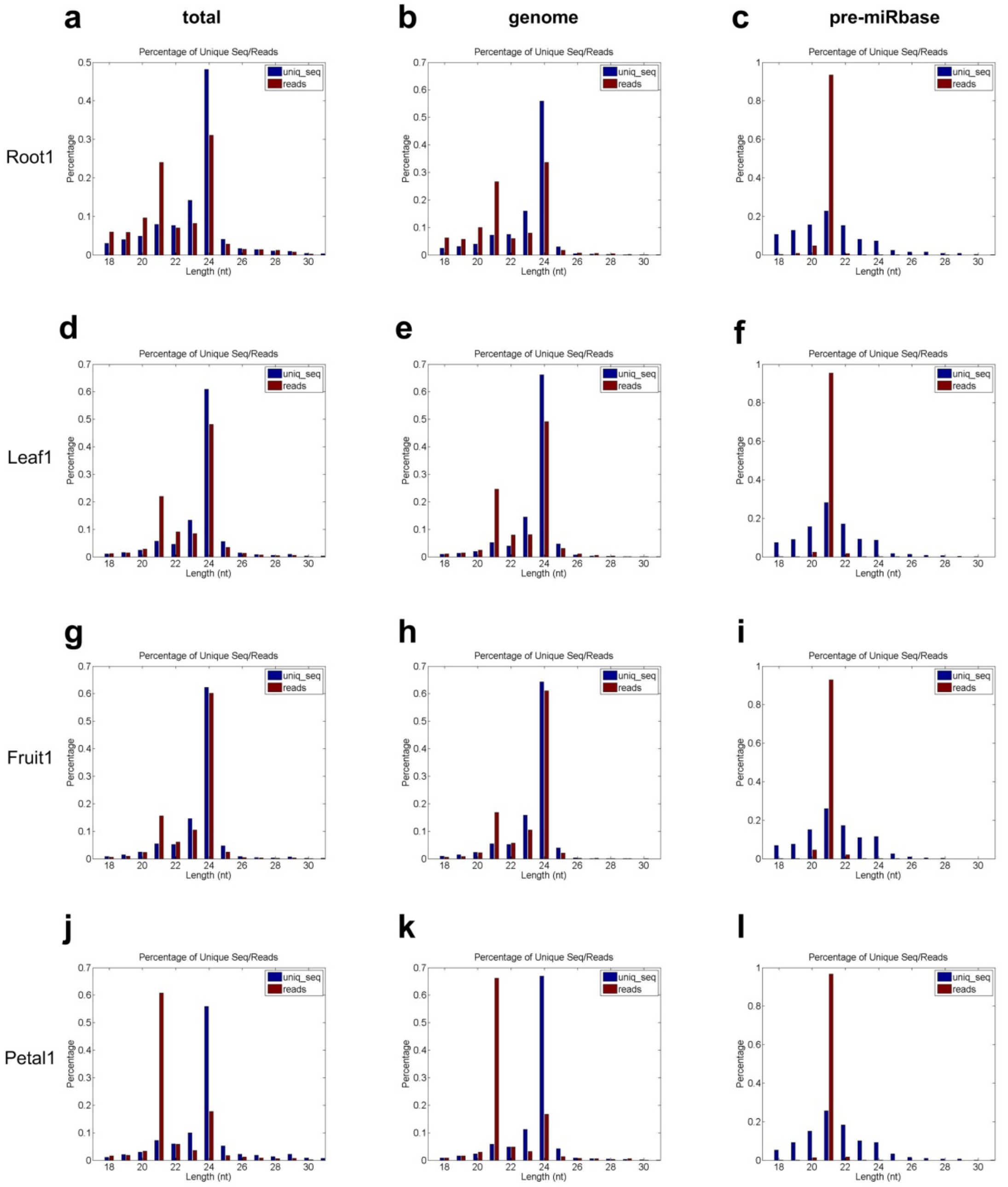

Figure S3. The length distributions of reads and unique sequences. The vertical axis shows the percentages of reads (red bars) and unique sequences (blue bars). The first column lists the distributions for reads and unique sequences in the whole libraries. The second column lists the distributions for reads and unique sequences mapped to watermelon genome. The third column lists the distributions for reads and unique sequences mapped to the pre-miRBase. (a) - (c) The distributions for the first root sample. (d) - (f) The distributions for the first leaf sample. (g) - (i) The distributions for the first fruit sample. (j) - (l) The distributions for the first petal sample.

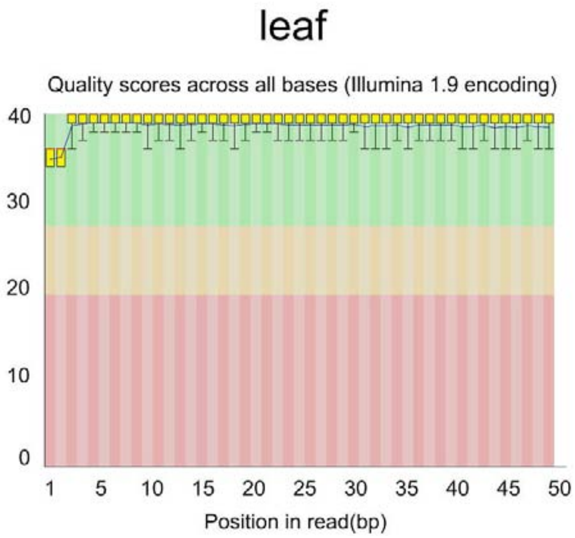

Figure S4. Per base sequencing quality of degradome sequencing profile. The horizontal axis is the position of the reads. The vertical is the quality scores of the reads.

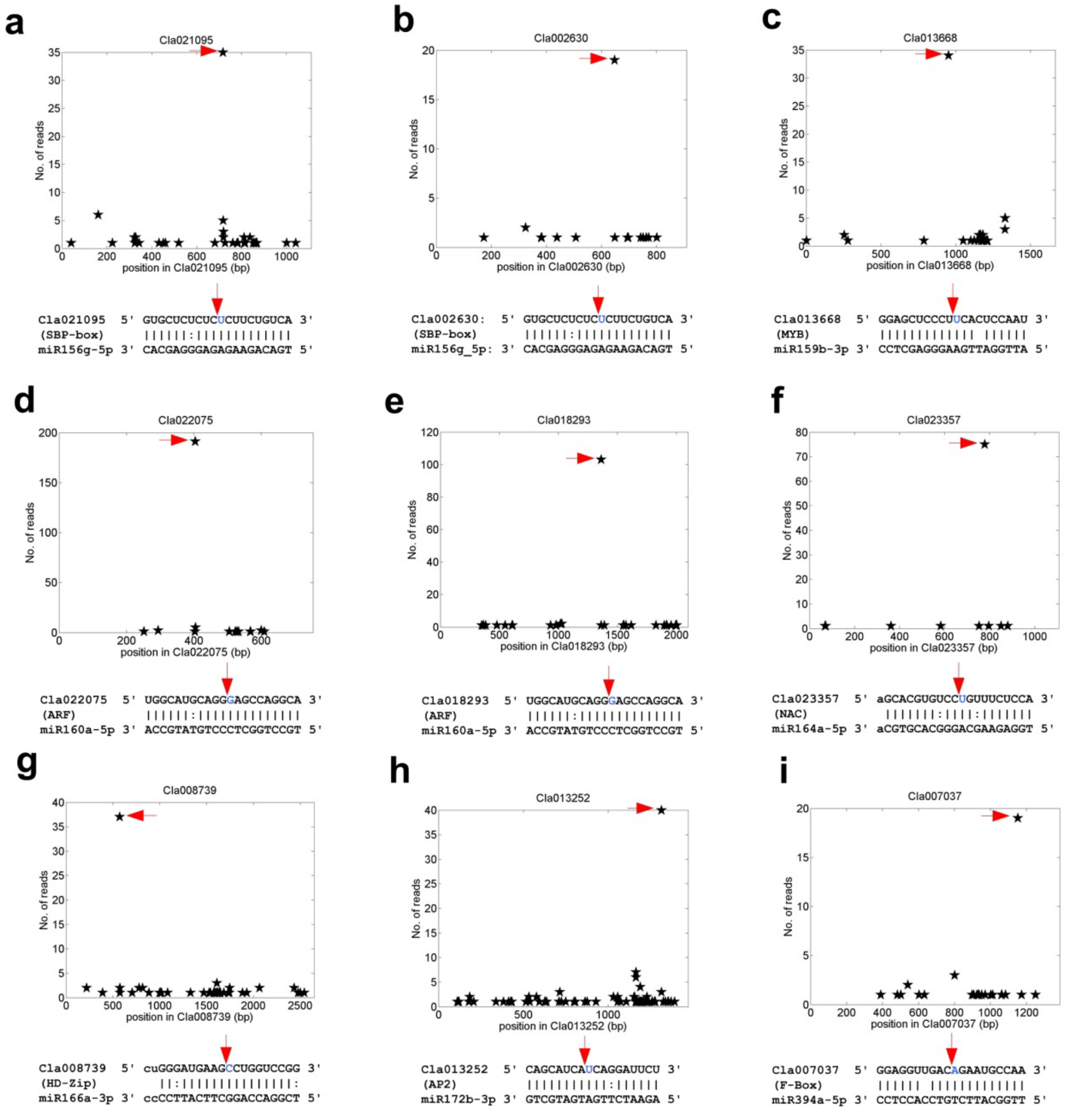

Figure S5. Some of the identified conserved miRNA targets. The x-axis is the position on the transcript, and y-axis is the number of reads detected from a position. The arrows in the upper parts correspond to the positions pointed by the arrows of the same colors in the lower parts. (a) miR156g-5p:Cla021095, an SBP transcription factor gene. (b) miR156g-5p:Cla002630, an SBP transcription factor gene. (c) miR159b-3p:Cla013668, a MYB transcription factor gene. (d) miR160a-5p :Cla022075, an ARF gene. (e) miR160a-5p:Cla018293, an ARF gene. (f) miR164a-5p:Cla023357, a NAC transcription factor gene. (g) miR166a-3p:Cla008739, an HD-Zip gene. (h) miR172b-3p:Cla013252, an AP2 transcription factor gene. (i) miR394a-5p:Cla007037, an F-box gene.

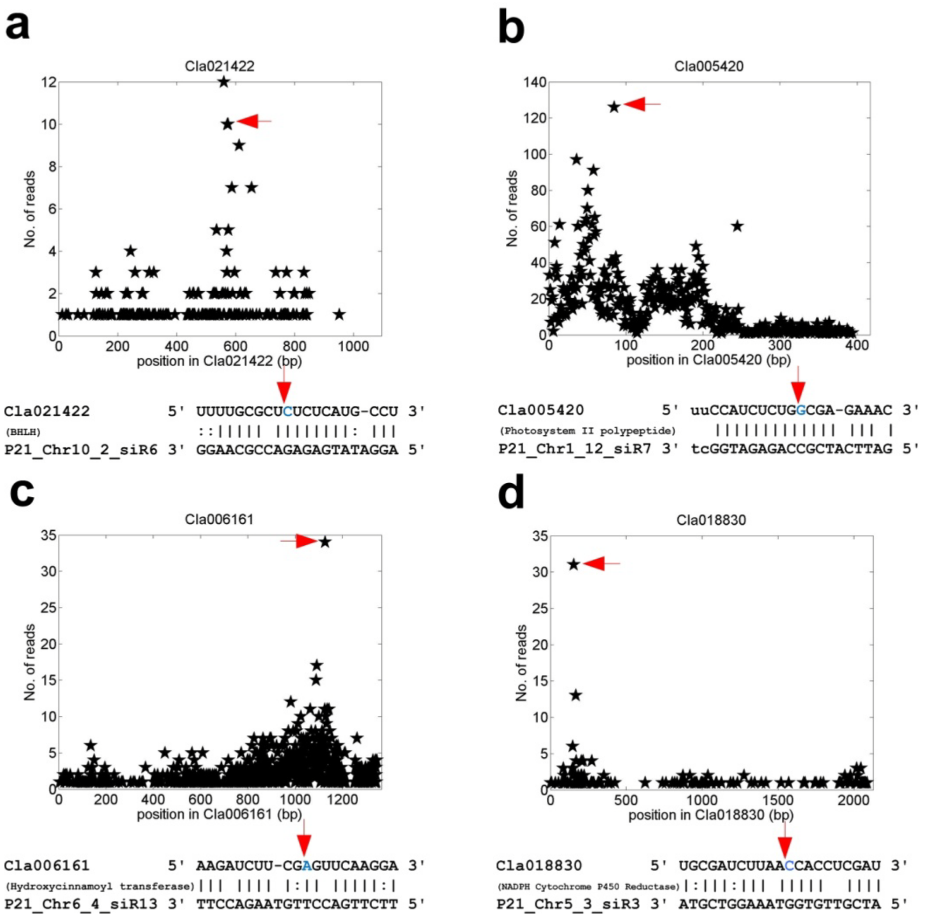

Figure S6. Some of the identified phasiRNA targets. The x-axis is the position on the transcript, and y-axis is the number of reads detected from a position. The arrows in the upper parts correspond to the positions pointed by the arrows of the same colors in the lower parts. (a) P21_Chr10_2_siR6:Cla021422, a BHLH transcription factor (b) P21_Chr1_12_siR7:Cla005420, a photosystem II polypeptide. (c) P21_Chr6_4_siR13:Cla006161, a Hydroxycinnamoyl transferase. (d) P21_Chr5_3_siR3:Cla018830, a NADPH-cytochrome P450 reductase.
